# Colloidal Gelation Under Dynamic Perturbation: A Departure from Classical Behavior

**DOI:** 10.3390/gels11120985

**Published:** 2025-12-08

**Authors:** Bin Xia, Xiaorong Wang

**Affiliations:** 1 School of Chemical Science and Engineering, Tongji University, Shanghai 200092, China; 2 Center for Frontier Research & Technology, Zhongce Rubber Group Co., Ltd., Hangzhou 310018, China

**Keywords:** colloidal gels, shear frequency, gelation, the liquid-to-solid transition, jamming, rheology

## Abstract

This study investigates the influence of dynamic perturbation on gelation behavior in a model colloidal system composed of hydrophobic silica particles dispersed in dioctyl phthalate. Contrary to the prevailing assumption that gelation is independent of oscillatory frequency, particularly at small strain amplitudes within the linear viscoelastic regime, our results reveal a pronounced dependence of gelation dynamics on the frequency of applied shear. In contrast, variations in strain amplitude and shear rate amplitude exert minimal effects. This observed behavior deviates significantly from classical gelation theory, which typically predicts frequency-independent rheological properties at the gel point. The results uncover a previously unrecognized viscoelastic phenomenon in soft colloidal materials, wherein microstructural rearrangements near the gelation threshold appear to be modulated by the timescale of mechanical excitation. As a result, traditional criteria for identifying gelation become less effective. The liquid-to-solid transition in these colloidal systems aligns more closely with the physics of particle jamming, rather than polymer network formation.

## 1. Introduction

Colloidal gels are soft solid materials characterized by an interconnected network of submicrometer-sized particles that spans the entire volume of the dispersion medium [1,2,3,4,5,6,7,8]. This particle network is transient in nature and can be disrupted or fluidized under shear deformation. In many cases, once the shear is removed, the disrupted network can automatically reconstruct. When the particle concentration is sufficiently high, this recovery process may occur within minutes or even seconds [9,10,11]. Consequently, the formation of the particle network can be regarded as the final arrested state of the colloidal system. Due to their inherent structural stability, colloidal gels are widely utilized in foods, pharmaceuticals, cosmetics, and paints. Their physical properties are primarily governed by the size and shape of the dispersed particles, the nature of interparticle interactions, the characteristics of the dispersion medium, and the spatial uniformity of the particle network [1,2,3,4,5,6,7,8,9,10,11,12].

A variety of methods have been proposed to identify the gel point [13,14,15,16,17,18,19,20,21,22,23,24,25,26] in colloidal systems, i.e., the moment at which a colloidal system transitions from a viscous liquid to an elastic solid. This sol–gel transition signifies the onset of solid-like behavior and plays a pivotal role in both fundamental understanding and practical applications. Accurate determination of the gelation point is essential for enabling precise control over the structural evolution of colloidal materials, mechanical properties, and overall performance. Common techniques include: (1) Tilt or inversion test [13], which assesses gelation visually by the cessation of flow under gravity; (2) Elastic modulus threshold [15,16,20], where a sudden rise in elastic modulus signals gelation; (3) Viscosity divergence [16,17,18,19], in which the steady shear viscosity approaches infinity; (4) Crossover method [14,15,20], which detects the point when storage modulus *G*′ exceeds loss modulus *G*″; (5) Winter-Chambon method [24,25], where gelation occurs when the damping factor tan*δ* becomes frequency-independent.

Among these methods, the tilt test is the most straightforward, though its qualitative nature limits accuracy. Methods relying on viscosity divergence or abrupt changes in modulus often require extrapolation and may be affected by singularities near the transition point [15,16,17,18,19,20]. In contrast, dynamic rheometric techniques can provide continuous, quantitative assessment throughout the gelation. Consequently, dynamic rheometers have become standard tools for tracking liquid-to-solid transitions and characterizing the properties of mature gels [14,15,21,22,23,24,25,26]. These measurements typically employ small strain amplitudes (≤1%) to ensure that the response of the test material remains within the linear viscoelastic regime, thereby preserving the integrity of the microstructure [24,25,26].

Given the increasing reliance on dynamic rheometers to determine sol–gel transition behavior, it is important to clarify the role of oscillatory frequency in this context. Although high-frequency oscillations could conceivably influence the material’s microstructure, mechanical destruction is generally considered to be negligible during gel point identification, provided the strain amplitudes remain sufficiently low and within the linear viscoelastic regime. This assumption appears to be well-supported from both theoretical and experimental perspectives. Theoretically, it draws from the principles of critical phenomena and the physics of percolation networks in soft matter, which predict that the gel point exhibits universal characteristics that are independent of frequency variation [27]. Experimentally, when frequency assignments are evenly spaced and strain amplitudes are carefully maintained within the linear regime, the crossover point of tan*δ* consistently identifies the gelation point with high robustness, regardless of the specific frequency values [24,25,26].

However, in the case of certain physically bonded systems, such as colloidal suspensions, emulsions, creams, and dairy products, dynamic rheometric techniques sometimes yield inconsistent results compared to chemically bonded systems [14,15,23,28,29,30]. These materials exhibit complex behavior near the gelation point, making the gelation point determination feel more like a subjective art or personalized methodology than a rigorously reproducible scientific process [14,15]. As a result, the perceived universality of gelation physics becomes obscured. Although several explanations have been proposed [15], the foundational assumption of whether the classical rheological theory of gelation can still be applied to colloidal systems with finite attractive interactions and reversible aggregation kinetics has rarely been critically examined.

This study examines the validity of this assumption mentioned above. Utilizing a binary colloidal system composed of hydrophobic silica particles suspended in a synthetic organic oil, we observed a striking deviation. The gelation process of a colloidal suspension resembles the dynamics of a physically jammed particle system rather than that of an interconnected polymer network. Here, we present a detailed analysis of this departure behavior and briefly discuss its implications for advancing the fundamental understanding of gelation phenomena in colloidal systems.

## 2. Results and Discussion

**Figure 1:**
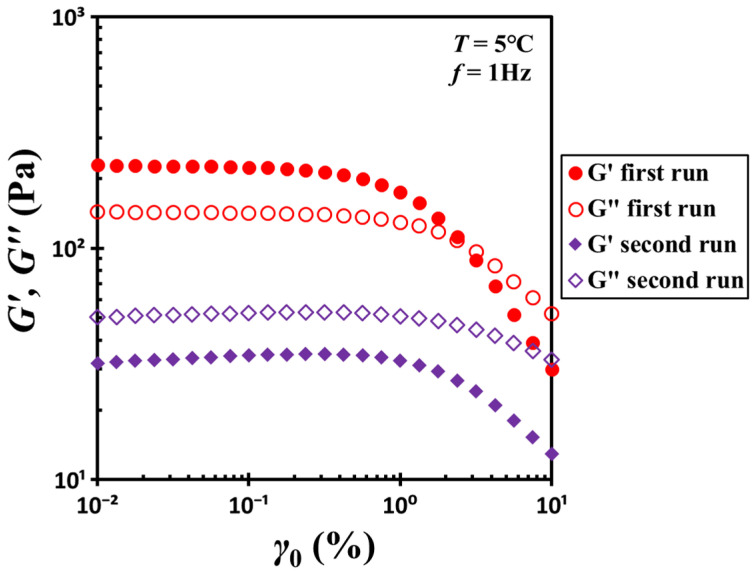
Strain dependence of the storage modulus *G*′ and loss modulus *G*″ for the G17/DOP binary suspension with a fixed filler content *ϕ* = 0.04. Red open circles and filled circles represent the initial strain-sweep measurements, while purple hollow diamonds and filled diamonds correspond to subsequent strain-sweep measurements conducted after initial shear. Test conditions: *f* = 1 Hz and *T* = 5 °C.

Figure 1 shows the rheological properties of a hydrophobic silica/DOP gel containing 10.4% silica by weight (volume fraction *ϕ* = 0.04). At 5 °C, the initially loaded material exhibits solid-like behavior, as its storage modulus *G*′ exceeds its loss modulus *G*″. However, this solid-like response is maintained only under small deformations. As the strain amplitude increases, *G*′ decreases beyond the yielding strain *γ_y_* (≈1%). As the strain further increases, *G*′ and *G*″ intersect, after which *G*′ continues to decline and falls below *G*″. A subsequent strain-sweep test conducted immediately after the first run confirms that *G*′ remains consistently lower than *G*″ throughout the entire strain range, indicating a full transition to fluid-like behavior.

**Figure 2:**
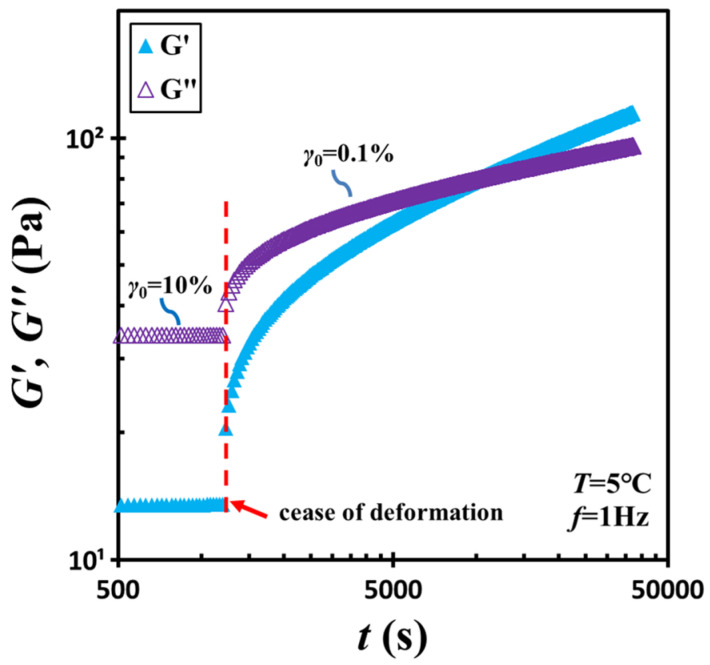
Low-strain storage modulus *G*′ and loss modulus *G*″ measured following a sudden change of strain amplitude from 10% to 0.1%. The arrow marks where the deformation changes. The test material is a G17/DOP binary suspension with a fixed filler content of *ϕ* = 0.04. The test conditions: *f* = 1 Hz and *T* = 5 °C.

To examine structural recovery, the material was subjected to shear at 10% strain amplitude and a frequency of 1 Hz for an additional 20 min. Subsequently, the strain amplitude was abruptly reduced from 10% to 0.1%, and an oscillatory time-sweep test was conducted to monitor the temporal evolution of viscoelastic properties. Here, a strain amplitude of 0.1% was selected to ensure measurements remained within the linear viscoelastic regime while still generating sufficient torque signals. The result is shown in Figure 2. Immediately following shear cessation, the material displayed liquid-like characteristics, evidenced by *G*″ > *G*′. As time increased, both moduli increased, with *G*′ rising more rapidly than *G*″. Their intersection occurred at a characteristic time (i.e., *t_c_* = 9000 s), beyond which the material transitioned back to a solid-like state as *G*′ surpassed *G*″.

**Figure 3:**
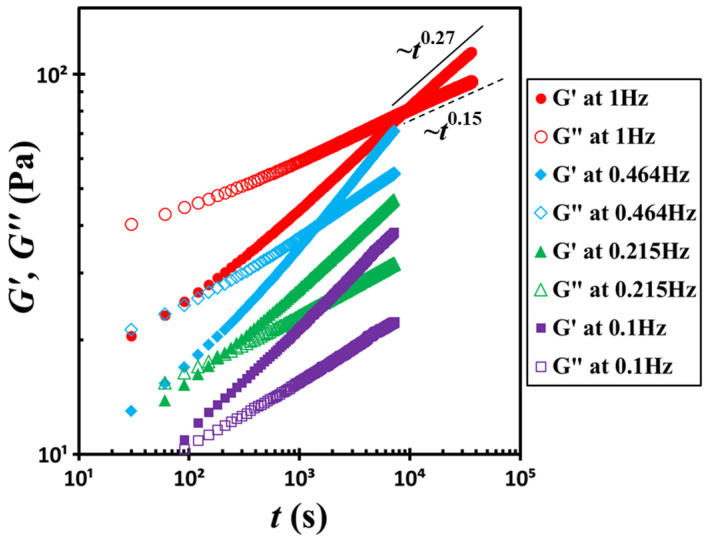
Temporal evolution of storage moduli *G*′ and loss moduli *G*″ at four different frequencies, 0.1, 0.215, 0.464, and 1 Hz. The solid line and dashed line represent fits to power-law functions. The test material is a G17/DOP binary suspension with a fixed filler content of *ϕ* = 0.04. The test conditions: *γ*_0_ = 0.1% and *T* = 5 °C.

Figure 3 presents similar data for the same suspension measured at four different frequencies, 0.1, 0.215, 0.464, and 1 Hz. In this plot, the time origin was redefined as the moment of shear cessation. At all frequencies, *G*′ and *G*″ follow power-law scaling relationships with time *t*: *G*′~*t*^0.27^ and *G*″~*t*^0.15^. However, the crossover time between *G*′ and *G*″ varies significantly with frequency. At *f* = 0.1 Hz, the crossover occurs at *t_c_* = 60 s, while at *f* = 0.464 Hz, the crossover shifts to *t_c_* = 1470 s. Furthermore, at *f* = 1 Hz, the crossover is delayed to *t_c_* = 9000 s. This crossover time marks the onset of structural arrest in the suspension under specific test conditions. Apparently, increasing the probe frequency prolongs the crossover time. Notably, the dramatic shift from 60 s to 9000 s over a relatively modest change in frequency (approximately one order of magnitude) is unexpected and prompts a critical question: Is this pronounced effect primarily governed by the probe frequency itself, or is it a consequence of the associated strain amplitude or shear rate amplitude?

**Figure 4:**
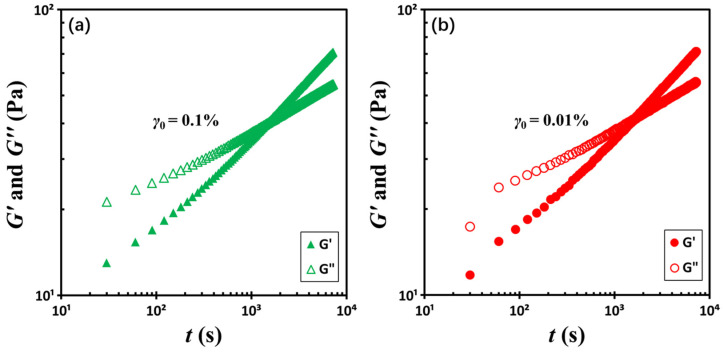
Temporal evolution of storage moduli *G*′ and loss moduli *G*″ at a constant frequency of 0.464 Hz under two strain amplitudes: (**a**) 0.1% and (**b**) 0.01%. The test material is a G17/DOP binary suspension with a fixed filler content of *ϕ* = 0.04 and measurements were conducted at a temperature of 5 °C.

To investigate the role of strain amplitude *γ*_0_ or shear rate amplitude ${\dot{\gamma }}_{0}$, two time-sweep experiments were then performed at a constant frequency of 0.464 Hz but with differing strain amplitudes: 0.1% and 0.01%. According to the relationship ${\dot{\gamma }}_{0}=\gamma_{0}\omega$, a tenfold increase in strain amplitude results in a corresponding tenfold increase in shear rate amplitude. Figure 4 presents a comparison of the two datasets. For clarity, the two datasets are presented in separate figures, grouped by strain level, to prevent the appearance of overlap. Despite the significant change in shear rate, the crossover times remain relatively consistent, measured at *t_c_* = 1470 ± 60 s for *γ*_0_ = 0.1%, and *t_c_* = 1560 ± 60 s for *γ*_0_ = 0.01%. This marginal discrepancy contrasts sharply with the over two orders of magnitude difference in crossover times observed in Figure 3, suggesting that strain amplitude or shear rate amplitude is not the dominant parameter. Rather, test frequency appears to be the principal factor governing the crossover point.

Nevertheless, it should be noted that the crossover point between the storage modulus *G*′ and the loss modulus *G*″ should not be regarded as a definitive criterion for determining the gel point. Tung and Dynes [23] observed that in chemically bonded polymer gels, when the frequency increases tenfold, the frequency dependence of the crossover point increases by about 1 min. Winter [26] found that this crossover point occurs near the gel point of chemically bonded gels, but not exactly at the gel point. He and his coworkers [24,25,26] proposed that a reliable experimental methodology of the critical threshold should be the frequency-independence of the loss tangent tan*δ*.

**Figure 5:**
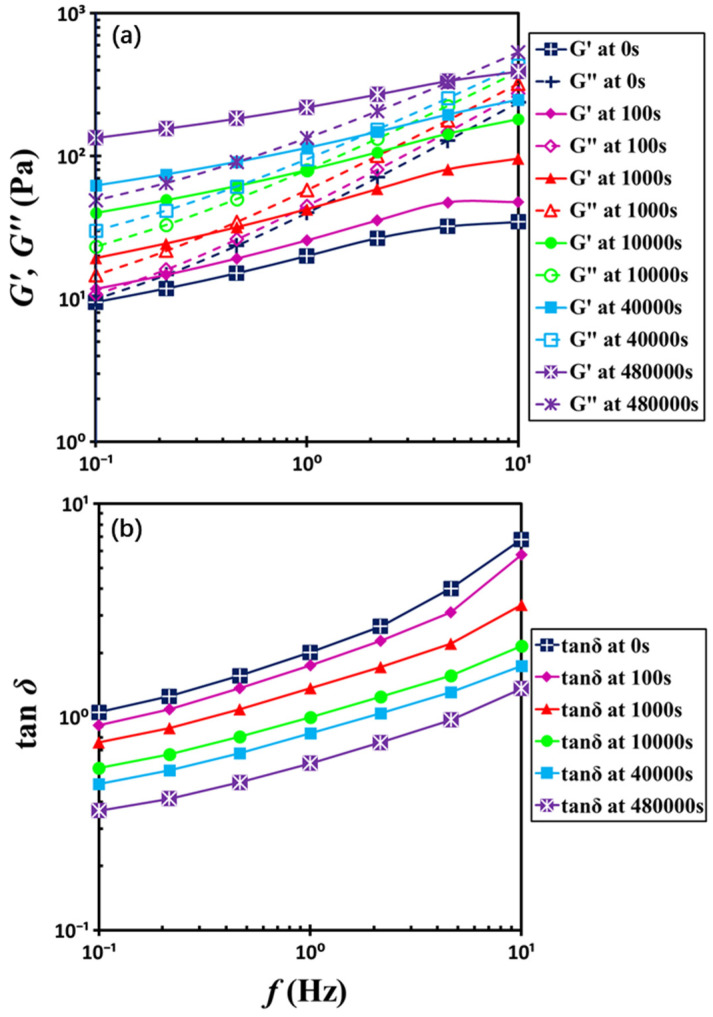
Frequency dependences of (**a**) dynamic moduli *G*′ and *G*″ and (**b**) loss factor tan*δ* with the indicated waiting times. The test material is a G17/DOP binary suspension with a fixed filler content of *ϕ* = 0.04. The test conditions: *γ*_0_ = 0.1% and *T* = 5 °C.

In order to determine the gelation point, frequency sweep measurements were conducted along the temporal evolution shown in Figure 4, with a frequency range from 10 Hz to 0.1 Hz. These measurements are like snapshots in time [24,25,26], enabling continuous tracking of the change in viscoelastic properties, thereby providing the rheological features of the transition from liquid to solid behavior. Figure 5 illustrates the frequency sweep results for six distinct waiting times. At low rest times, *G*″ remains higher than *G*′ across most of the frequency range, consistent with liquid-like characteristics. At extended waiting times during gelation, however, the system exhibits *G*′ surpassing *G*″, reflecting the emergence of solid-like characteristics. Intriguingly, at high frequencies (e.g., *f* = 10 Hz), the loss modulus *G*″ exceeds the storage modulus *G*′, indicating a predominantly liquid-like response, whereas at lower frequencies *G*′ surpasses *G*″, signifying solid-like behavior. As gelation progresses, the dynamic moduli *G*′ and *G*″ increase over time and the crossover point of *G*′ and *G*″ shifts toward higher frequencies, revealing unique dynamic microstructural evolution during the solidification.

The observed viscoelastic crossover phenomenon represents a notable departure from the classical rheological behavior typically exhibited by polymers. In conventional polymer melts or solutions, the dynamics are governed by chain relaxation processes [31], resulting in elastic or solid-like behavior (*G*′ > *G*″) at high frequencies and liquid-like terminal flow (*G*″ > *G*′) at low frequencies. Similarly, physically crosslinked gels [32] also display liquid-like behavior at low frequencies due to the reversible nature of their physical crosslinks, which can break and reform over time. When subjected to sufficiently low-frequency deformation, these dynamic bonds have plenty of time to reorganize, leading to a fluid-like response.

The colloidal gel examined in this study is likewise a physically bonded system, characterized by a percolated network of submicrometer-sized attractive particles that spans the entire volume of the dispersion medium. However, it exhibits an inverted frequency-dependent trend. Unlike typical polymer gels, this colloidal system behaves as an elastic solid at rest and transitions to a fluid state only under sufficient mechanical excitation. This inverted frequency-dependent behavior suggests that the particulate microstructure manifests a unique rheological signature, where solid-like behavior dominates at low frequencies due to the stability of the network, and yielding occurs only when the applied stress disrupts the structure.

Supporting evidence from the literature includes the work of Trappe et al. [33], who examined several attractive colloidal systems and observed similar inverted frequency dependence during the sol–gel transition. They attributed this phenomenon to the concept of “jamming,” which shares fundamental physical principles with glass formation [34]. Consequently, the inverted viscoelastic trend observed during the liquid-to-solid transition can be rationalized within this framework. However, the implications of such anomalous behavior on classical rheological theory of gelation were not addressed in their analysis.

The inverted frequency dependence observed during the sol–gel transition leads to a marked deviation in the behavior of the loss factor, tan*δ*, from the classical rheological definition of the gel point. As illustrated in Figure 5b, tan*δ* for the colloidal gel increases monotonically with frequency across all temporal snapshots. This trend contrasts sharply with classical gelation theory [35], which typically describes a decreasing, concave-up profile of tan*δ* prior to the gel point, a frequency-independent plateau at the gel point, and an increasing, concave-down trajectory thereafter. The persistent concave-up increase in tan*δ* throughout the sol–gel transition suggests that relaxation mechanisms have been evolved throughout the frequency-sweep process, reflecting the dynamics of a physically jamming system of particles [2] rather than that of an interconnected polymer gel. Consequently, the classical framework of gelation fails to adequately capture the rheological characteristics of this colloidal system.

**Figure 6:**
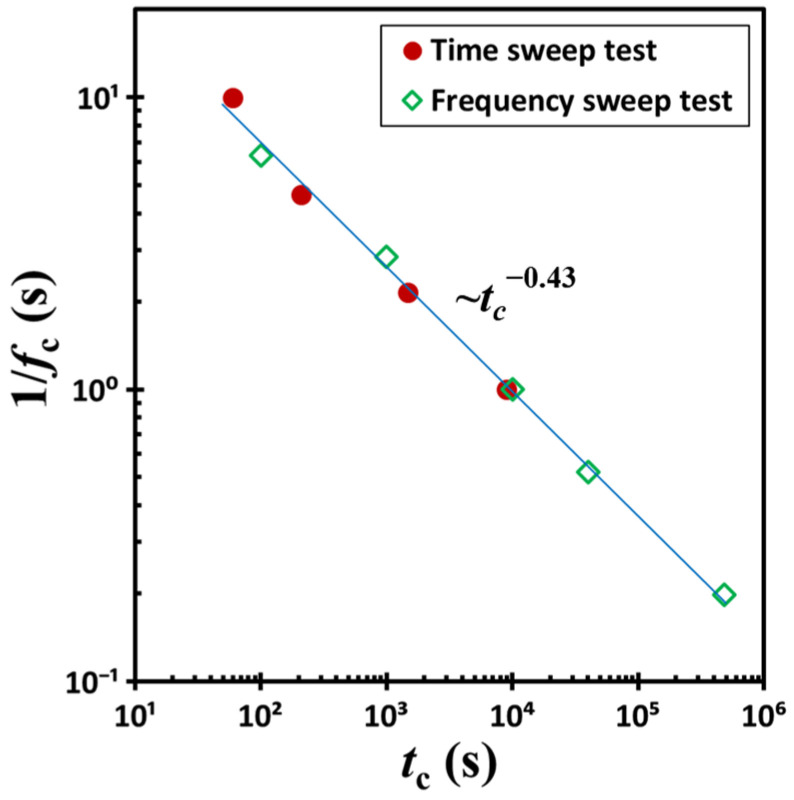
The inverse of the sampling frequency (i.e., the period 1/*f_c_*) plotted as a function of time *t_c_*. Data were obtained from time-sweep tests conducted at varying shear frequencies and from frequency-sweep experiments performed at different waiting times. The test material is a G17/DOP binary suspension with a fixed filler content of *ϕ* = 0.04. The test conditions: *γ*_0_ = 0.1% and *T* = 5 °C.

By extracting the crossover time *t_c_* and corresponding shear frequency *f_c_* from Figure 3 and Figure 5 at the point where the storage modulus *G*′ and loss modulus *G*″ intersect, we constructed Figure 6 to further analyze the data. In this framework, the reciprocal of *f_c_* defines the oscillation period: *p_c_* = 1/*f_c_*. When plotted on a dual logarithmic scale, a clear linear correlation emerges between *p_c_* and *t_c_*, derived from both time-sweep experiments at varying shear frequencies and frequency-sweep experiments at differing waiting times. Again, samples subjected to longer waiting periods exhibit proportionally lower values of *p_c_*. This trend follows a simple power-law relationship: *p_c_*∼*t_c_*^−0.43^. According to this scaling, *p_c_* tends toward infinity as *t_c_* approaches zero, implying that the corresponding shear frequency *f_c_* approaches zero.

However, experimental validation of this extrapolation remains challenging, as it lies beyond the practical limits of current measurement techniques. Nonetheless, visual observations reveal that the colloidal suspension swiftly regains its gel-like state following the cessation of shear deformation induced by vigorous stirring. In particular, a tilt test at 23 °C demonstrates that structural recovery occurs within a minute, suggesting rapid reformation of the gel network in the absence of mechanical disturbance. Nevertheless, it should be noted that Figure 6 reveals a striking result: a two-order-of-magnitude change in excitation frequency *f* leads to an approximately five-order-of-magnitude shift in the crossover time *t_c_*.

To interpret the unexpected frequency dependence of the liquid-to-solid transition behavior, it is essential to consider the inherent tendency of attractive particles in a liquid medium to agglomerate and form a jammed network. The transition from a liquid-like to a solid-like state is likely governed by the collective dynamics of the dispersed particles. When structural rearrangements within the system occur on timescales comparable to those of the imposed oscillatory shear, the external mechanical perturbation can significantly influence the formation and evolution of the particle network. This interplay between intrinsic particle motion and applied frequency may explain the observed rheological response.

A rough estimate of the characteristic frequency of Brownian motion of filler particles [36,37] can be derived from the Stokes-Einstein relation: *D*/*a*^2^ = *kT*/6π*ηa*^3^, where *T* is the temperature, *k* is the Boltzmann constant, *η* is the viscosity of the medium, *D* is the diffusion coefficient of a colloidal particle, and *a* is the particle radius. In this colloidal suspension, the mobile entities are fumed silica aggregates, with diameters on the order of several hundred nanometers. Considering the viscosity of the dispersion medium (or DOP) at 5 °C is approximately 0.22 Pa·s, the estimated frequency (*D*/*a*^2^) of particle motion falls within the range of 1 to 10 Hz. Remarkably, this range aligns closely with the shear frequencies employed during rheological measurements. The overlap of these timescales implies that structural rearrangements of filler particles may occur at a rate comparable to that of the applied mechanical perturbation.

We anticipate that this phenomenon may be prevalent across a range of colloidal systems, including emulsions, creams, and dairy products, where the sizes of dispersed particles typically span from submicrometers to several micrometers. The present findings underscore the potential relevance of particle-jamming physics in shaping the formulation and processing behavior of such systems, particularly in dairy applications. Accordingly, the observed deviations from classical theoretical predictions in certain colloidal food systems [14,15,23,28,29,30] may arise from a similar underlying mechanism, where particle dynamics and externally applied mechanical excitation are intertwined together. This interplay offers a plausible explanation for these discrepancies frequently reported in the literature.

**Figure 7:**
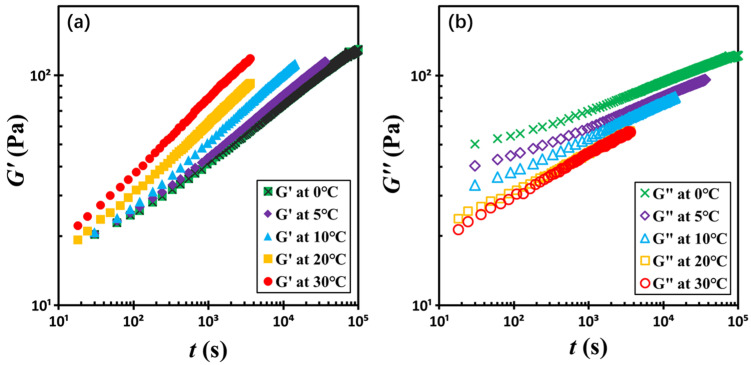
Temporal evolution of (**a**) storage moduli *G*′ and (**b**) loss moduli *G*″ with the indicated temperatures. The test material is a G17/DOP binary suspension with a fixed filler content of *ϕ* = 0.04. The test conditions: *γ*_0_ = 0.01% and *f* = 1 Hz.

To assess the influence of temperature on the rheological behavior of silica-filled colloidal gels, we investigated the time-dependent evolution of the dynamic moduli *G*′ and *G*″ at 1 Hz across a temperature range of 0 to 30 °C. For clarity, five representative datasets at seven different temperatures are presented in Figure 7. Remarkably, the moduli evolution curves obtained at different temperatures can be collapsed onto a single master curve through a two-step normalization process. First, *G*′ and *G*″ are normalized by their respective critical values *G_c_*′ and *G_c_*″ at the crossover point. Second, the time axis is rescaled by the critical crossover time *t_c_*, yielding the reduced time scale *t*/*t_c_*. This approach enables a unified picture of structural recovery dynamics across various temperatures.

**Figure 8:**
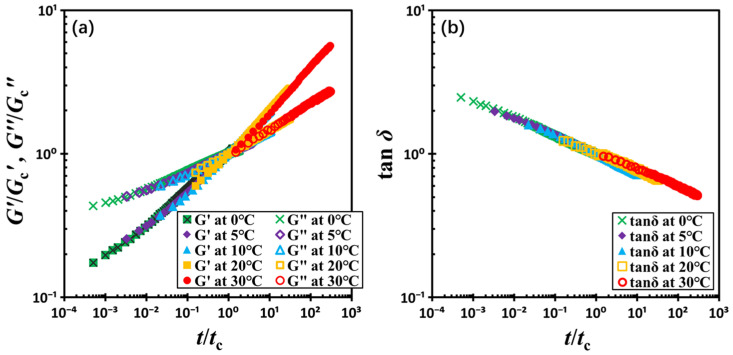
(**a**) Normalized moduli *G*′/*G_c_*′ and *G*″/*G_c_*″ and (**b**) loss factor tan*δ* as a function of normalized time *t*/*t_c_*. The test material is a G17/DOP binary suspension with a fixed filler content of *ϕ* = 0.04. The test conditions: *γ*_0_ = 0.01% and *f* = 1 Hz.

Figure 8 illustrates the resulting master curve. In the regime where *t*/*t_c_* < 1, the material exhibits liquid-like behavior, characterized by *G*′ < *G*″. Conversely, for *t*/*t_c_* > 1, the material transitions into a solid-like state with *G*′ > *G*″. The point *t*/*t_c_* = 1 signifies the onset of kinetic arrest, marking the transition from a viscous fluid-like state to an elastic solid-like state. This temperature-independent master curve behavior suggests that, within the experimental range, the kinetics of gel formation in this colloidal system is primarily dominated by the relative time evolution rather than the absolute temperature.

**Figure 9:**
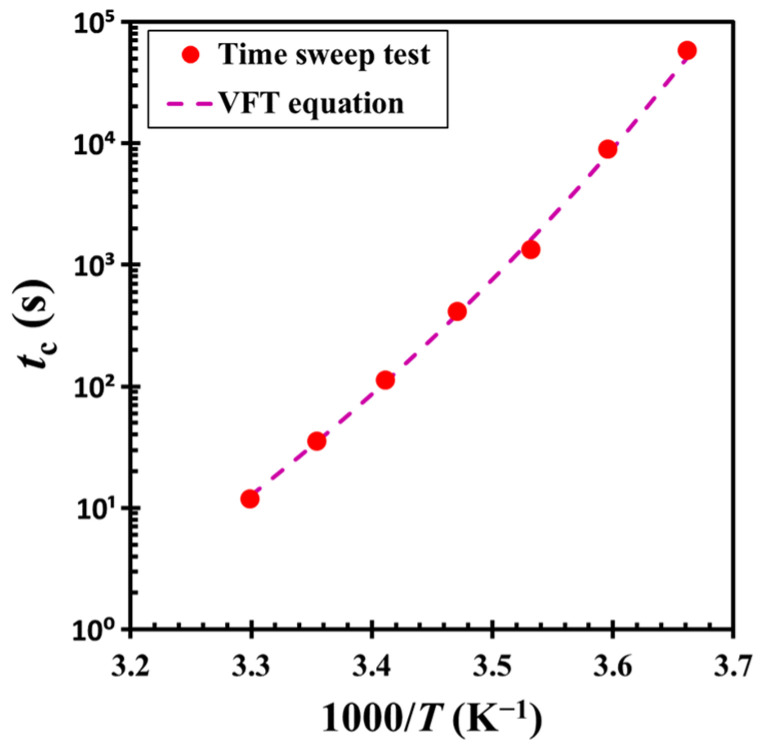
Critical crossover time *t_c_* as a function of inverse temperature 1/*T*. The dashed line represents a fit to the experimental data using the Vogel-Fulcher-Tammann (VFT) equation: *t*_c_ = 2.20 × 10^−8^ exp [2074.84/(*T* − 200.22)]. The test material is a G17/DOP binary suspension with a fixed filler content of *ϕ* = 0.04. The test conditions: *γ*_0_ = 0.01% and *f* = 1 Hz.

The master curve analysis indicates that the material undergoes gelation via a consistent kinetic mechanism across different temperatures. However, the critical crossover time *t_c_* displays a pronounced temperature dependence, as shown in Figure 9. This relationship is well described by the Vogel-Fulcher-Tammann (VFT) equation [38]: *t_c_* = *A* exp[*B*/(*T* − *T*_0_)], where *A* = 2.20 × 10^−8^ s and *B* = 2074.84 K, and *T*_0_ = 200.22 K represents the Vogel-Fulcher-Tammann temperature. The equation captures the marked deceleration of gelation dynamics as the temperature approaches *T*_0_ from above, consistent with the experimental observations. Notably, *T*_0_ is close to the glass transition temperature of DOP (*T_g_* = −85.75 °C or 187.40 K), suggesting that the dispersion medium plays a significant role in the gelation process. As the temperature approaches *T_g_*, the viscosity of the medium increases dramatically, significantly limiting particle mobility. This suppression of particle motion near *T_g_* results in a significant extension of the crossover time, underscoring the critical influence of medium viscosity on the kinetics of structural evolution.

**Figure 10:**
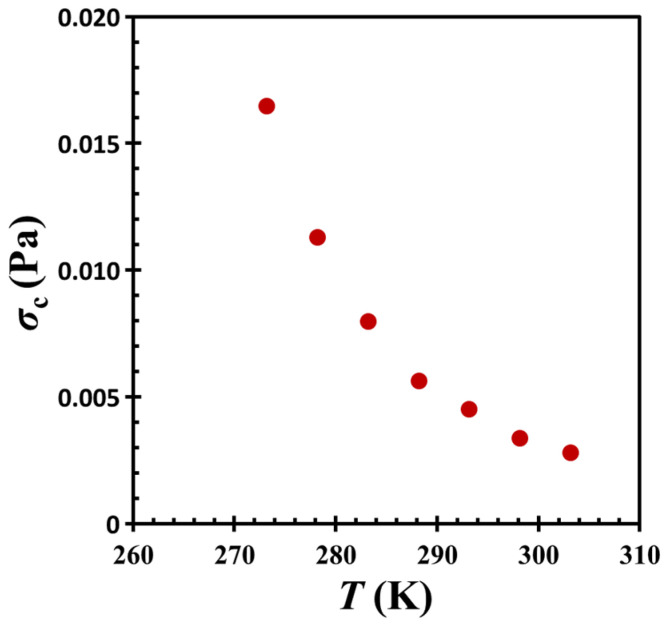
Temperature *T* vs. critical stress *σ_c_* for G17/DOP binary suspension with a fixed filler content of *ϕ* = 0.04. The test conditions: *γ*_0_ = 0.01% and *f* = 1 Hz.

We notice that in the related literature [2,33,34], shear stress *σ* is usually employed to characterize the jamming phase diagram. In the present study, the maximum stress is defined by *σ* = │G*│*γ*_0_, where G* is the complex modulus of the material and *γ*_0_ is the strain amplitude. This equation allows that the critical stress *σ_c_* (= │G_c_*│*γ*_0_) at the crossover point (or jamming transition) can be estimated accordingly. Figure 10 depicts the relationship between temperature *T* and critical stress *σ_c_* for the colloidal gel under investigation. Notably, the behavior observed in the oil-hydrophobic silica particle mixture remains consistent with the iso-concentration plane of the jamming phase diagram reported by Trappe et al. [33], following a monotonic concave trajectory. This observation further supports the interpretation of the colloidal system as a gel governed by particle jamming dynamics.

**Figure 11:**
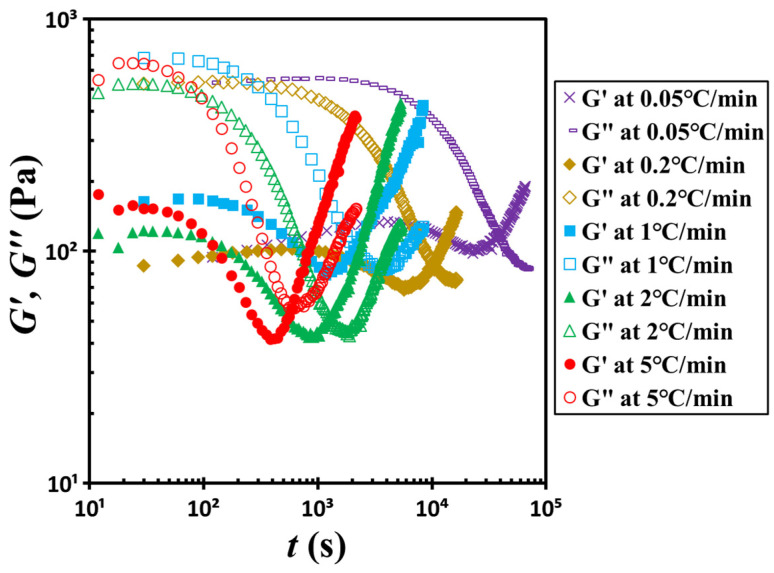
Evolution of the dynamic moduli *G*′ and *G*″ as a function of time during the non-isothermal temperature ramp-up experiments conducted at various heating rates ranging from 0.05 to 5 °C/min. The test material: G17/DOP binary suspension with a fixed filler content of *ϕ* = 0.04. The test conditions: *γ*_0_ = 0.01% and *f* = 1 Hz.

To further verify the influence of temperature on the sol–gel transition behavior, we conducted a series of non-isothermal temperature ramp-up experiments. Figure 11 presents the time-dependent evolution of the storage modulus *G*′ and loss modulus *G*″ under heating rates ranging from 0.05 to 5 °C/min. At the beginning of each test, the material displays liquid-like rheological characteristics, with *G*″ significantly exceeding *G*′. As the temperature increases, both moduli gradually decline, with *G*″ decreasing more rapidly than *G*′. Eventually, the curves reach their respective minima and then intersect at a characteristic crossover time *t_c_*. Beyond this point, both *G*′ and *G*″ begin to rise, with *G*′ increasing more sharply, which signals a transition to solid-like behavior as *G*′ surpasses *G*″.

**Figure 12:**
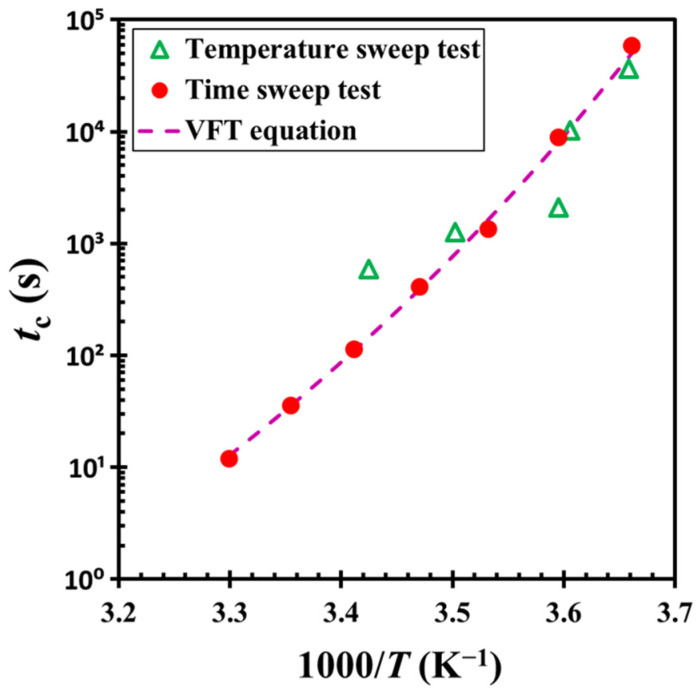
Critical crossover time *t_c_* as a function of inverse temperature 1/*T*. The plot compares *t_c_* values obtained from non-isothermal temperature ramp-up experiments and isothermal time sweep tests. The test material: G17/DOP binary suspension with a fixed filler content of *ϕ* = 0.04. The test conditions: *γ*_0_ = 0.01% or 0.1% and *f* = 1 Hz.

Importantly, the crossover time *t_c_* shortens with increasing heating rate, indicating that higher temperatures accelerate the structural evolution associated with the gelation. We extracted the *t_c_* values from these ramp-up experiments and incorporated them into Figure 9 for comparison. A summary of these results is provided in Figure 12. As shown, the temperature dependence of *t_c_* under non-isothermal conditions follows the same trend predicted by the VFT relationship. However, the data exhibit greater scatter in this case compared to those obtained under isothermal conditions. This variability is likely due to the discrepancies between the rheometer’s thermal chamber temperature and the actual sample temperature, which may not be uniform throughout the non-isothermal testing process.

**Figure 13:**
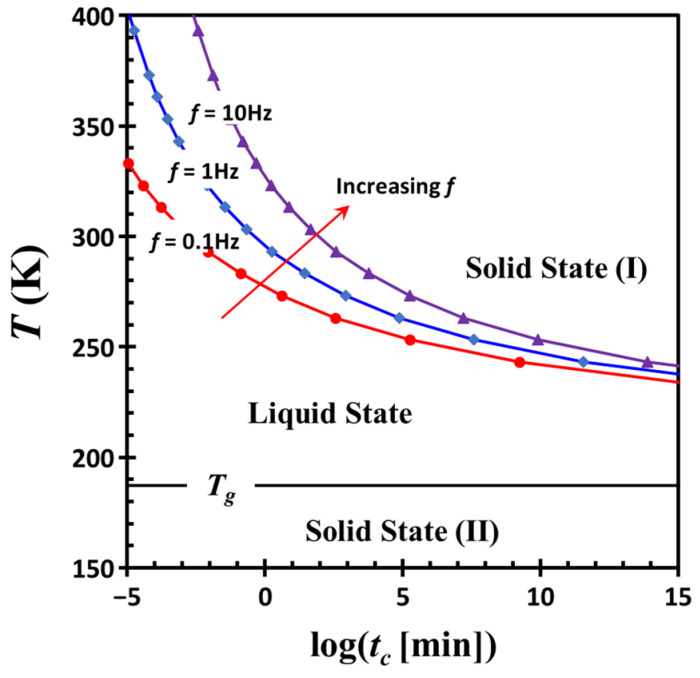
Time-temperature transformation diagram calculated using the empirical relationship: *t_c_* = 2.20 × 10^−8^ *f*^1/0.43^ exp [2074.84/(*T* − 200.22)]. The diagram illustrates the transition between liquid-like and solid-like states as a function of time, temperature, and excitation frequency. Solid state (I) corresponds to the formation of an interconnected network of colloidal particles within the dispersion medium, while solid state (II) represents the vitrification of the medium itself. The liquid state lies between these two regimes. The calculations are based on experimental data obtained after cessation of severe oscillatory shear deformation in a G17/DOP binary suspension with a fixed filler content *ϕ* = 0.04.

The combined effects of shear frequency *f* and temperature *T* on the crossover time *t_c_* are well captured by the following empirical relationship: *t_c_* = 2.20 × 10^−8^ *f*^1/0.43^ exp [2074.84/(*T* − 200.22)]. Using this equation, a time-temperature transformation diagram [39] is constructed (Figure 13), illustrating the transition between liquid-like and solid-like states as a function of time, temperature, and excitation frequency. This diagram highlights several key features, including the glass transition temperature *T_g_* of the dispersion medium, commonly referred to as the vitrification point, and the boundaries between liquid and solid regions. The diagram also reflects the frequency-dependent nature of colloidal network structures, distinguishing two types of solid-like behavior. Solid state (I) refers to the formation of an interconnected network of colloidal particles within the dispersion medium, while solid state (II) corresponds to the vitrification of the medium itself. The liquid state lies between these two states. It is a non-equilibrium state and its existence range strongly depends on temperature and frequency. The phase boundary between the liquid state and solid state (I) is highly sensitive to excitation frequency, shifting upward with increasing frequency. In contrast, the boundary between the liquid state and solid state (II) remains relatively stable, governed by the molecular-level vitrification of DOP. Importantly, the solidification process of the latter occurs independently of the formation of a colloidal particle network.

This framework offers a conceptual basis for understanding the physical behavior of colloidal systems, in which the liquid-to-solid transition results from interparticle interactions, external perturbations, and the properties of the dispersion medium. By mapping the rheological transformations that occur under varying temperature, time, or frequency conditions, the diagram may serve as a valuable tool for optimizing the design of colloidal systems, as many of which support commercially important products and everyday applications. Further exploration of this diagram across different materials may yield deeper insights into their rheological characteristics and design potential.

## 3. Conclusions

This study investigates the frequency-dependent gelation behavior of a model colloidal system composed of hydrophobic silica particles dispersed in dioctyl phthalate (DOP). Notably, the material consistently exhibits an inverted viscoelastic spectrum: it behaves as a solid (*G*′ > *G*″) at low frequencies and transitions to a fluid-like state (*G*″ > *G*′) at high frequencies. This behavior is more characteristic of a physically jammed particle system than of a chemically or physically interconnected polymer gel. We attribute this inversion to the structural rearrangement of filler particles occurring on a timescale comparable to that of the applied mechanical perturbation. In contrast to classical gelation theory, which predicts frequency-independent rheological properties at the gel point, we have observed that this gel has a pronounced sensitivity to oscillatory frequency. Meanwhile, variations in strain and shear rate amplitudes exert minimal influence within the linear viscoelastic regime. These findings highlight the critical role of oscillation frequency in governing the evolution of viscoelastic properties and gelation dynamics in such systems. To further elucidate these transitions, we introduce a time-temperature transformation diagram that maps the boundaries between liquid-like and solid-like states as functions of time, temperature, and excitation frequency. This framework provides a clear means of distinguishing rheological behavior under varying thermal and mechanical conditions and offers conceptual relevance to other colloidal suspensions and gels, particularly those containing large dispersed particles.

## 4. Materials and Methods

### 4.1. Materials and Preparation

**Figure 14:**
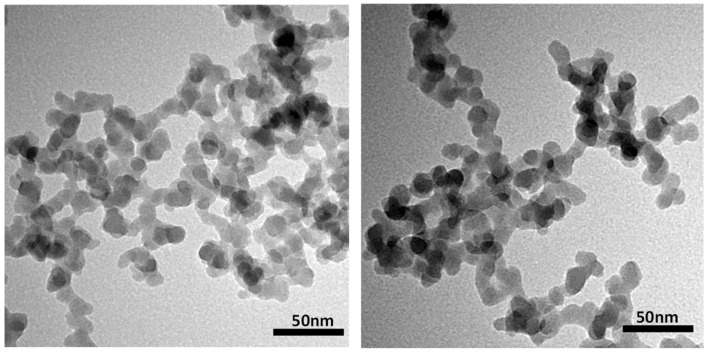
TEM microscopic images of hydrophobic silica G17 particles.

The hydrophobic nano-silica used in this study was a product numbered G17, which came from Changtai Micro-Nano Chemical Factory in Shouguang City, China. The diameter of the primary particles ranges from 10 to 30 nanometers, with an average size of about 20 nanometers. Transmission electron microscopy (JEOL JEM-2000, Tokyo, Japan) shows that the primary particles of G17 form aggregates of approximately a hundred nanometers in diameter with various spatial configurations and three-dimensional branched structures (Figure 14). The diameters of these aggregates were measured using a dynamic light scattering (DLS) analyzer (Anton Paar Litesizer 500, Graz, Austria), and the average hydrodynamic diameter of the aggregates was found to be 101.06 nm.

**Figure 15:**
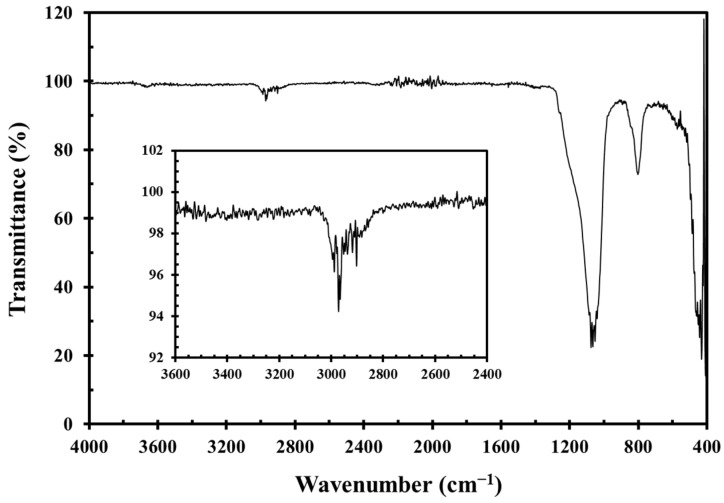
FT-IR spectrum of hydrophobic silica G17 particles. The inset shows the characteristic absorption of methyl groups around 2950 cm^−1^.

According to the manufacturer, the particle surfaces were treated with hexamethyldisilazane during production. This treatment, which involves reacting hexamethyldisilazane with silanol groups on the silica surface at elevated temperatures, is one of the standard industrial practices for producing hydrophobic silica. The active nitrogen atoms in hexamethyldisilazane promote thorough methylation of the silica during high-temperature treatment [40,41]. Infrared spectroscopy (Thermo Fisher Scientific Nicolet iS10, Madison, WI, USA) confirmed the absence of surface silanol absorption (typically near 3480 cm^−1^) in G17 and revealed strong methyl group absorption around 2950 cm^−1^ (Figure 15), indicating a surface methylation degree of ≥95%. The density of G17 silica is 2.6 g/cm^3^.

**Figure 16:**
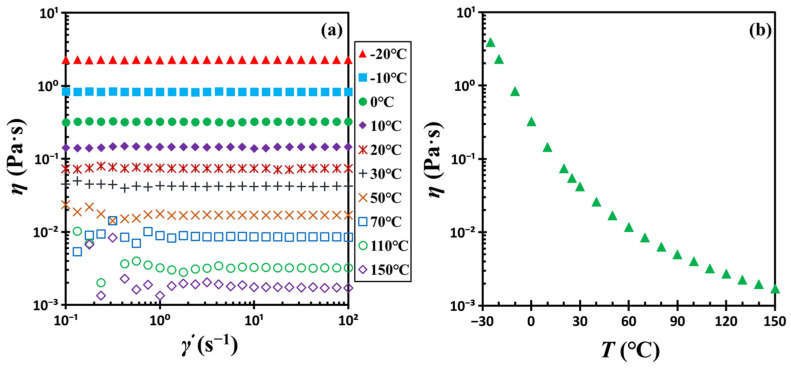
(**a**) Steady shear viscosity *η* of DOP vs. shear rate $\dot{\gamma }$ and (**b**) steady shear viscosity *η* of DOP vs. temperature *T*.

The dispersion medium was dioctyl phthalate (DOP), a synthetic organic oil or plasticizer of 99% purity, purchased from Aladdin Chemical Reagents Co., Ltd. (Aladdin, Shanghai, China). Its well-defined molecular structure distinguishes it from polymeric substances. DOP is a stable, non-volatile liquid suitable for experiments within the −30 to 150 °C range. Its glass transition temperature (*T_g_*) was determined to be −85.75 °C (or 187.40 K) via differential scanning calorimetry (DSC). Figure 16 shows the temperature-dependent viscosity of DOP, measured with a rotational rheometer. DOP behaves as a Newtonian fluid over this temperature range, with shear-independent viscosity that increases rapidly as the temperature decreases. Tetrahydrofuran (THF) was also 99% pure and obtained from Aladdin. All materials were utilized in their as-received condition.

Colloidal gels consisting of G17 silica and DOP were prepared by dispersing both in excess tetrahydrofuran (THF), followed by solvent removal. In the preparation, a predetermined mass of silica was placed in a flask. Then, THF of approximately ten times the silica mass was added to the flask. The mixture was stirred at 300 rpm for 2 h using a magnetic stir bar (8 mm diameter, 20 mm length) to ensure that the silica particles were well dispersed in the THF. The dispersion was then placed in an ice-water bath and was ultrasonicated at 70 W for another 30 min to break up particle aggregates. Subsequently, a predetermined mass of the DOP oil was added and mixed for 12 h under gentle stirring. Once the silica was well dispersed, the mixture was poured into a flat pan to form a thin liquid film of a few millimeters in thickness. The volatile THF was first removed under a draft hood and then further degassed in a vacuum oven at 25 °C for 72 h to eliminate residual THF. The final material was collected into glass bottles and further vacuum-treated at 25 °C for 24 h prior to testing.

### 4.2. Measurements

Viscoelastic properties of the colloidal gels were evaluated using an Anton Paar EC Twist 302 stress-controlled rheometer with torque ranging from 0.02 μNm to 200 mNm. This rheometer was controlled by a standard RHEOPLUS 3.2 software in a waveform oscillatory test setup. At frequencies below 10 Hz, this stress-controlled rheometer can be operated effectively in strain-controlled mode. Temperature control was achieved using a P-PTD200 Peltier base plate and an H-PTD200 forced-convection hood (Anton Paar, Graz, Austria), allowing operation from −40 °C to 200 °C with ±0.1 °C accuracy.

Measurements were performed using a cone-plate geometry (50 mm plate diameter, 0.04 rad cone angle). A flat plate fixture (50 mm diameter) was also sometimes employed for convenience. Geometry variation did not impact results herein, as tests remained within the linear viscoelastic regime. In preparation, the colloidal sample to be tested was typically loaded onto the lower plate at 23 °C with more material in the center of the plate than at the edges. When the upper plate was lowered, the first contact of the sample with the upper plate would be in the center of the plate, thereby minimizing the possible trapping of air between the material and the plate. After the material was loaded between the fixtures, it was cooled down to the test temperature, such as 5 °C. Upon thermal equilibrium attainment, two strain amplitude sweep tests were conducted at a constant shear frequency of 1 Hz, with strain amplitudes progressively increased from 0.01% to 100%. This procedure was designed to disrupt the material’s internal structure through large-amplitude shear deformation.

In the measurements, the material was first subjected to pre-shearing at 10% strain amplitude and 1 Hz for 20 min to ensure any filler network was disrupted and the material completely transformed into a stable liquid state. Then, a time-sweep test was performed at 0.1% strain and a selected frequency ranging from 0.1 to 1 Hz to monitor the temporal evolution of viscoelastic properties. The time origin was defined as the moment of large shear cessation. Once the material reached a specific rheological stage, a frequency sweep test was sometimes conducted at 0.1% strain amplitude (like a snapshot), with a frequency range from 10 Hz to 0.1 Hz, to characterize the frequency response after the material had evolved.

For temperature ramp-up experiments, the material was first pre-sheared at 10% strain and −30 °C for 20 min. Then, under low-strain oscillation shear condition (0.01% strain, 1 Hz), the sample temperature was ramped from −30 °C to 150 °C at different rates ranging from 0.05 to 5 °C/min. Owing to the prolonged duration required to reach 150 °C at the slower heating rates of 0.05 and 0.2 °C/min, the tests were stopped upon observing a transition from liquid to solid behavior. To minimize the potential of shear or temperature-induced property alterations, a fresh sample was always loaded before initiating each new test.

Wall slip was evaluated by applying Fourier transform analysis to the original waveform data. In cases where wall slip occurs, the even harmonic coefficient *I*_2_/*I*_1_ typically exhibits a sudden and pronounced increase. All measurements reported in this study were conducted within the linear viscoelastic regime at low strain amplitudes. Upon inspection, the values of *I*_2_/*I*_1_ consistently remained below 1%. Accordingly, wall slip and its potential influence on the rheological measurements are negligible in this work.

## Data Availability

The data presented in this study are openly available in article.
